# Proteases at work: cues for understanding neural development and degeneration

**DOI:** 10.3389/fnmol.2015.00013

**Published:** 2015-05-05

**Authors:** Paul Saftig, Paola Bovolenta

**Affiliations:** ^1^Institut für Biochemie, Christian-Albrechts-Universität zu Kiel, KielGermany; ^2^Centro de Biología Molecular “Severo Ochoa", Consejo Superior de Investigaciones Científicas – Universidad Autónoma de Madrid, MadridSpain; ^3^Centro de Investigación Biomédica en Red de Enfermedades Raras, MadridSpain

**Keywords:** neurogenesis, axon guidance, synapse formation, stem cells, ADAM, presenilin, neurodegeneration, prion

## Abstract

Proteolytical processing of membrane bound molecules is a fundamental mechanism for the degradation of these proteins as well as for controlling cell-to-cell communication, which is at the basis of tissue development and homeostasis. Members of families of metalloproteinases and intra-membrane proteases are major effectors of these events. A recent workshop in Baeza, Spain, was devoted to discuss how this mechanism coordinates brain development and how its dysfunction leads to brain pathologies. Herein we summarize the findings presented during this workshop, which illuminate the role of metalloproteinases, including matrix metalloproteinase, A Disintegrin and Metalloproteinase-proteases and intra-membrane proteases, in the regulation of neurogenesis, axon guidance, and synaptogenesis as well as in neurodegeneration. Indeed, there is increasing evidence that proteolysis at the membrane is directly linked to neuropathologies such as Alzheimer Disease and autism spectrum or prion disorders. These proteolytic events are tightly regulated and we are just at the beginning of understanding how these processes could be exploited to design therapeutic treatments aimed at alleviating psychiatric and neurodegenerative pathologies.

## Introduction

The highest functions of the nervous system, including the cognitive capabilities of the human brain, are rooted on stereotyped and yet plastic interactions among a huge variety of neurons and glial cells, which compose the vertebrate brain. The specification of neural cells occurs with an apparently invariable precision during a protracted period of the embryonic and early postnatal life (Albright et al., 2000). In turn, the molecular information acquired by the different neuronal types is fundamental to select and restrict the establishment of brain circuit and to modify the way in which information is processed (Zipursky and Sanes, 2010). All these events need to be highly coordinated in time and space with mechanisms that control how the flow of information among cells is switched off or prolonged. Therefore, understanding how the human brain is built requires the full comprehension of this diversity and of the mechanisms that finely control the formation and the posterior plasticity of neuronal circuits (Emes and Grant, 2012). This, in turn, is a prerequisite to identify the molecular basis of the abnormalities associates with the many disorders that affects the different structures of the brain and their function.

Proteolytical processing of membrane bound molecules acquires particular importance in the context of the brain since its function strongly depends on well-orchestrated interactions among many different cell types, including neurons and glial cells. Members of families of metalloproteinases are major effectors of these events. The meeting “Proteases at work: cues for understanding neural development and degeneration” which took place in Baeza, Spain from October 20th until October 23rd, 2014 brought together internationally recognized leaders in the field to summarize the most recent evidence demonstrating that proteases are essential for the development of the CNS, underscoring their relevance in neurodegeneration and discussing whether endogenous or pharmacological modulation of their activity represents a therapeutic tool.

**FIGURE 1:**
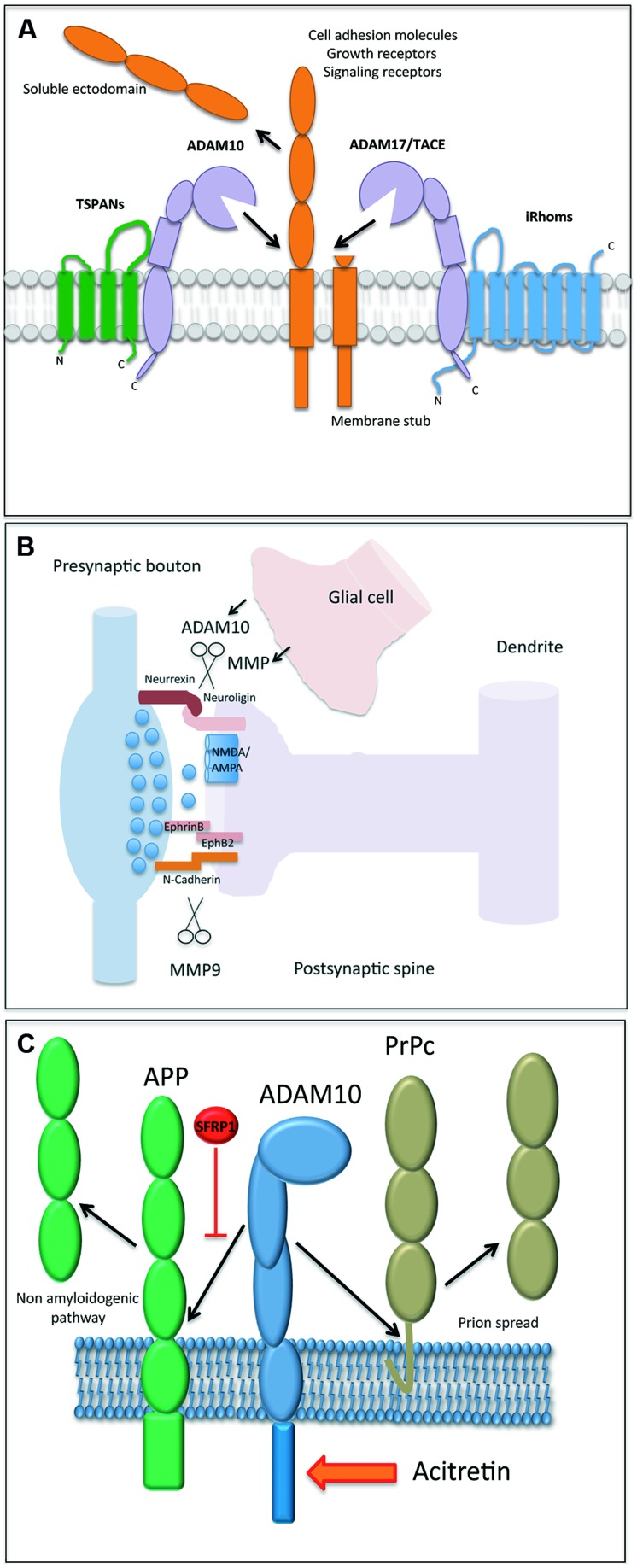
**(A)** Illustration of an A Disintegrin and Metalloproteinase (ADAM) protease, which is regulated by tetraspanin proteins (e.g., shown for ADAM10) and inactive rhomboids (shown for ADAM17). The cleavage of a membrane bound substrate protein is a hallmark of the process termed “ectodomain shedding.” It regulates the function of this cell surface protein and its half-life. **(B)** Proteases play a central role in both excitatory and inhibitory synapse biology and function. ADAM10 and MMP9 may contribute to synaptic plasticity by the proteolytic processing of essential synaptic adhesion molecules, such as neuroligins and cadherins. Proteases expressed in glial cells may modulate (*in trans*) the shedding events on postsynapses as well. **(C)** The processing of amyloid precursor protein (APP) and the cellular prion protein (PrPc) is an essential element in the molecular pathogenesis of Alzheimer and Prion Disease, respectively. The reported identification of physiological (Sfrp1) and pharmacological (acitretin) modulators of their proteolytical processing adds new perspective to protease function.

*Carl Blobel* (Hospital for Special Surgery, New York, NY, USA) initiated the conference by providing an overview about proteinases belonging to the A Disintegrin and Metalloproteinase Family (ADAMs). ADAMs regulate the fate of cell surface expressed membrane proteins (Blobel, 2005; Weber and Saftig, 2012; **Figure 1**). He focused on a specific member of this family, ADAM17, and its well-established role in liberating Epidermal Growth Factor Receptor ligands from their membrane-anchor. As for the other proteolytically active members of the ADAM family, their regulation still remains a field with many open questions. In this regard, the inactive Rhomboid1 and 2 (iRhom) proteins turned out to be instrumental upstream regulators for ADAM17/EGFR signaling. iRhom2 controls the substrate selectivity of stimulated ADAM17-dependent ectodomain shedding (Maretzky et al., 2013). Its interaction with ADAM17 controls the cellular trafficking of the protease and its maturation in myeloid cells (**Figure 1A**). Disruption of both *iRhoms* genes in mice led to a phenotype comparable to the ADAM17 and EGFR-knockout mice providing clear evidence that ADAM17 is tightly controlled by both iRhoms.

## Metalloproteinases in Brain

Closely related to ADAM17, ADAM10 exerts a number of essential functions related to the development and function of the brain. ADAM10 sheds the amyloid precursor protein (APP), the Notch receptor, and the prion protein (Weber and Saftig, 2012). *Paul Saftig* (University Kiel, Germany) reported about a number of additional neuronal substrates of ADAM10. Conditional knockout mice with disruption of ADAM10 in the developing or adult brain revealed different and stage-dependent functions of this protease. ADAM10 controls cell fate decision events with a process that depends on the cleavage of the Notch receptor and that discriminates between neurogenesis and gliogenesis (Jorissen et al., 2010). In adult neurons, ADAM10 contributes to synaptogenesis mediating cleavage of adhesion proteins at the synapse (**Figure 1B**). Spine development, synapse plasticity, synaptic network function, memory, and learning all rely on proper cell-autonomous activity of ADAM10 (Prox et al., 2013). ADAM10 itself can be shedded by ADAM9 or ADAM15. It is thus conceivable that the glia-derived ADAM10-ectodomain could also contribute to synapse shaping *in trans*. Interestingly, trafficking of ADAM10 from the endoplasmic reticulum to its active site, the plasma-membrane, seems to be tightly controlled. Some members of the tetraspanin (TSPAN) family, including TSPAN15, associate with ADAM10 and are co-transported at the cell surface (Prox et al., 2012), where they apparently increase the life span of ADAM10 and help mediating substrate specificity (**Figure 1A**).

*Elena Marcello* from *Monica Di Lucaˊs* (University of Milan, Italy) laboratory also supported the idea that trafficking of ADAM10 is central to its regulation. The Synapse-Associated Protein-97 (SAP97) interacts with the cytoplasmic domain of ADAM10 (Marcello et al., 2007). This binding leads to transport of the protease to the post-synaptic membrane and in an Alzheimer Disease (AD) context causes an increased α-secretase cleavage of APP. The SAP97 contact to ADAM10 is further regulated by protein kinase C phosphorylation (Saraceno et al., 2014). Furthermore, the adaptor protein 2 (AP2) complex controls ADAM10 endocytosis, which is triggered by long-term-potentiation in hippocampal neurons, thereby reducing its surface shedding function. Of note, this ADAM10/AP2 association seems increased in AD brains (Marcello et al., 2013).

The synaptic role of ADAM10 was also the focus of *Taisuke Tomitaˊs* (University of Tokyo, Japan) presentation. The postsynaptic adhesion molecule neuroligin (NLG) binds to its presynaptic counterpart neurexin. Interestingly, NLG mutations have been linked to psychiatric disorders and mental retardation (reviewed in: Sudhof, 2008). ADAM10 cleaves NLG-1 after NMDA receptor activation. This causes an additional intra-membrane cleavage exerted by the γ-secretase complex (Suzuki et al., 2012). Dependent on neuronal stimulation, different members of the ADAM family may cleave different types of NLGs (**Figure 1B**). Therefore, ADAM proteases may be interesting targets to treat autism spectrum disorders, because, as mentioned above, NLGs have been strongly linked to these neuropsychiatric diseases (Sudhof, 2008).

The challenge for considering ADAM proteases as therapeutic targets will be the selective interference with the processing of one substrate, without considerably affecting other substrates, so to avoid undesired side effects. This is particularly important because over 65 neuronal substrates have been so far identified, as an example, for ADAM10. Some of these substrates were found using proteomic techniques in the conditioned medium of neurons derived from ADAM10 conditional knockout, as described by *Peer-Hendrik Kuhn* (Technical University Munich, Germany). This powerful approach not only validated a number of already described substrates but extended this list to previously unrecognized surface proteins involved in synapse function, axon guidance and intercellular adhesion. A similar approach previously and successfully identified new neuronal substrates for BACE-1, the β-secretase involved in APP processing (Kuhn et al., 2012).

Again related to synaptic function, *Leszek Kaczmarek* (Nencki Institute, Warsaw, Poland) described the pivotal importance of matrix metalloproteinase-9 (MMP-9) in the regulation of synaptic plasticity (**Figure 1B**), learning, and memory. MMP-9 belongs to the family of metzincins, which are enzymes secreted by neurons and glia (Yong et al., 2001) and, in contrast to ADAMs, their proteolytical activity occurs mainly in the extracellular space (**Figure 1B**). MMP-9 expression is tightly regulated and it is rapidly released near excitatory synapses in response to synaptic stimulation (Dziembowska et al., 2012). MMP9 cleaves the cell adhesion molecule nectin-3 under conditions of stress (van der Kooij et al., 2014) and its activity has been implicated in the synaptic alterations found in neuropsychiatric disorders, such as schizophrenia and autism (Tsilibary et al., 2014).

## Metalloproteinases in Development

Nervous system development depends on exquisitely coordinated cell communication, assuring that information among cells is activated where needed and switched off at or prolonged for the appropriate time. Controlled proteolysis contributes to this coordination, among others, by disrupting adhesion, modulating signaling and even regulating gene transcription, thereby influencing tissue patterning, morphogenesis and differentiation (Bai and Pfaff, 2011). *Shanthini Sockanathan* (Johns Hopkins University, Baltimore, MD, USA) illustrated this idea presenting a novel enzymatic mechanism, which controls Notch signaling, a classical example of a pathway activated by the generation of a transcriptionally active intracellular fragment (Pierfelice et al., 2011). Indeed, the differentiation of postmitotic neurons in the spinal cord depends upon the expression of GDE2, a six *trans*-membrane protein with an extracellular glycerophosphodiester phosphordiesterase (GDPD) domain (Sabharwal et al., 2011). GDE2 expressed by motorneurons enzymatically control the surface availability of the Notch ligand Delta1 in motor neurons, thereby promoting the differentiation of neighboring progenitors by decreasing Notch activity. GDE2 control of surface Delta occurs via a complex cascade that involves the GPI anchor cleavage of the ADAM10 repressor RECK (Park et al., 2013).

*Avraham Yaron* (Weizmann Institute of Science, Rehovot, Israel) instead discussed the relevance of proteolytical inactivation of axon guidance signaling mediated by the Neuropilin-1 (Nrp1) receptor and its Sema3A ligand. Sema3A acts as an axonal repellent for sensory axons projecting to the spinal cord. Genetic inactivation of ADAM10/ADAM17 causes guidance errors of proprioceptive axons, because the otherwise developmentally regulated shedding of Nrp1 at the sensory growth cones does not occur. This failed shedding abnormally prolongs the growth cone repulsive response to Sema3A. Accordingly, this repulsive response is no longer observed when growth cones express a form of Nrp1 highly susceptible to cleavage, further underscoring the importance of ectodomain shedding in switching growth cone responsiveness to guidance cues (Romi et al., 2014).

Still within the concept of proper axon growth regulation but this time of mouse retinal ganglion cells (RGC), *Paola Bovolenta* (Centro de Biología Molecular Severo Ochoa, CSIC-UAM, Madrid, Spain) presented data supporting that Secreted Frizzled Related Proteins (Sfrps), highly diffusible secreted modulators of cell–cell communication (Bovolenta et al., 2008), participate in mouse visual pathway development. Sfrps act by both directly signaling at the growth cone and negatively modulating ADAM-mediated proteolytical processing of other relevant cues (Marcos et al., 2015). These conclusions are consistent with previous observations demonstrating, on one side, that Sfrp1 reorients chick and *Xenopus* RGC growth cones (Rodriguez et al., 2005) and, on the other side, that Sfrp1 binds to ADAM10, down-regulating the processing of a number of its substrates, including *N*-cadherin or L1 (Esteve et al., 2011), which are involved in RGC guidance.

Adhesion regulation is crucial also to establish the circuitry in the developing cortex as discussed by *Patricia Maness* (University of North Carolina at Chapel Hill, NC, USA). Alterations in the function of GABAergic basket interneurons inhibiting pyramidal cells of the prefrontal cortex are thought to be responsible for working memory deficits in schizophrenia patients. On the other hand, ADAM-mediated proteolysis of NCAM is known to participate in synaptic plasticity during learning and memory (Brennaman et al., 2011). Taking the two observations together *Patricia* showed that excessive shedding of NCAM is associated with a decreased GABAergic basket cell function and an impaired working memory. This is because NCAM forms a complex with EphA3, which binds to ephrinA5 and mediates ADAM10-dependent elimination of basket cell synapses during postnatal development of the prefrontal cortex. Disruption of this complex increases basket cell perisomatic synapses and inhibitory responses in the prefrontal cortex, indicating that this regulatory mechanism may limit GABAergic inhibition in the cortex, establishing an appropriate excitatory/inhibitory balance (Brennaman et al., 2013, 2014).

As other organs, the brain maintains stem cell niches throughout adulthood. The mouse subependymal zone is one of these niches, in which a relatively quiescent stem population continually produces new neurons through the generation of rapidly diving, transit-amplifying progenitors (Porlan et al., 2013). *Isabel Fariñas* (University of Valencia, Spain) showed that *N*-cadherin-mediated adhesion to ependymocytes contributes to the quiescence of neural stem cells in the sub-ependymal niche. More interestingly, MT5-MMP, a membrane-type metalloproteinase, sheds *N*-cadherin ectodomain in this niche, ensuring the proper activation of the stem cell population in both physiological and regenerative conditions (Porlan et al., 2014), thus unveiling possible targets to stimulate neurogenesis when needed.

Following on the role of MMP, *Lieve Moons* (University Leuven, Belgium) demonstrated that two members of this family, MMP-2 and MMP-3, are required for the on-time migration of granule cells and GABAergic interneurons in the cerebellum as well as for a proper dendritic arborisation of the Purkinje cells and pyramidal neurons of the neocortex (Santiago-Medina et al., 2015). Notably, genetic inactivation of these MMP causes sustained deficits in motor performance in adult mice. Comparative proteomic approaches highlighted possible underlying molecular causes (Van Hove et al., 2015). Furthermore, as an interesting possibility linked to visual system repair, *Lieve* also discussed the potential implications of MMP-2 and MT1-MMP as promising axon-outgrowth promoting molecules within the CNS (De Groef et al., 2014; Gaublomme et al., 2014).

## Metalloproteinases in Neurodegeneration

γ-secretase-mediated intra-membrane proteolysis is a well-defined process, which involves regulated intra-membrane proteolysis of type-1 *trans*-membrane proteins. Since APP is a major substrate of the multi-protein γ-secretase complex and its cleavage contributes to the generation of amyloidogenic Aβ peptide species, inhibition of the γ-secretase has attracted much attention as a promising therapy for AD. *Bart de Strooper* (University Leuven, VIB, Belgium) discussed the possible causes of a failed phase III clinical trial using semagacestat, a γ-secretase inhibitor. Most likely owing to the inhibition of the Notch-1 signaling pathway, patients treated with this small compound presented serious side effects, including skin cancers. Treated AD patients were also subjected to infections and paradoxically experienced further cognitive decline (Doody et al., 2013). A possible explanation for this failure might be the potential need of lowering γ-secretase enzymatic activity moderately but chronically, a kinetic that might not have been achieved with the designed treating protocol. Rather, daily oscillations of the drug levels in the CSF might have caused periods of full Notch inhibition, exacerbating the side effects. Furthermore, the potential misprocessing of other γ-secretase substrates might have also contributed to these disappointing results (De Strooper, 2014). It is thus important to design a more selective inhibition of the γ-secretase complex capable of interfering with APP processing but not with that of other substrates (De Strooper and Chavez Gutierrez, 2015).

Related to the crucial need in Alzheimer research of developing effective therapies preventing the excessive generation of Aβ species and following the idea that Sfrp1 is one of the few soluble regulators of ADAM10, *Pilar Esteve* (Centro de Biología Molecular Severo Ochoa, CSIC-UAM, Madrid, Spain) showed that reduction of Sfrp1 expression shifts APP processing toward a non-amyloidogenic pathway (**Figure 1C**) in the adult mouse brain (Esteve et al., 2011). *Nigel M. Hooper* (University of Manchester, UK) instead showed that ADAM10 sheds PrP^C^ (Taylor et al., 2009), the cellular prion protein (**Figure 1C**), capable of binding, and mediating the toxicity of Aβ oligomers (Rushworth et al., 2013). This leads to the hypothesis that modulation of the shedding of PrP^C^ by ADAM10 would alter PrP^C^-Aβ oligomers’ interaction and thus their neurotoxicity. Data in this direction were presented, further supporting the idea that increasing ADAM10 activity may be beneficial for AD for multiple reasons. It will increase α-secretase processing of APP that reduces Aβ and at the same time increases sAPPα generation, thought to have neuroprotective properties, including the inhibition of Aβ production (Obregon et al., 2012). Furthermore, increased ADAM10-mediated shedding of PrP^C^ will buffer the toxicity of the remaining Aβ oligomers.

*Kristina Endres* (Clinic of Psychiatry and Psychotherapy, Johannes Gutenberg University, Mainz, Germany) raised several important considerations that need to be solved before pursuing ADAM10 as a therapeutic target. She underscored that it is still unclear to what extent a deficient ADAM10 catalytic activity or a reduced expression of its gene participates in AD pathogenesis. We also do not know the full network of factors regulating *ADAM10* expression in humans and whether pharmacological interference with ADAM10 enzymatic activity can be achieved in patients. More importantly, is it safe? *Kristina* reported encouraging results on both fronts with the identification of transcription factors that influence ADAM10 expression (Reinhardt et al., 2014). She also showed that acitretin, a synthetic retinoid, safely targets ADAM10 activity since AD patients treated with this compound presented a significant increase in the CSF levels of sAPPα, providing important basis to design larger and longitudinal trials to evaluate the possible clinical benefits of this treatment (Endres et al., 2014; **Figure 1C**).

The importance of metalloprotesases in neurodegeneration is not limited to AD or autism spectrum disorders and the case of another severely debilitating illness was illustrated. *Elena Cattaneo* (University of Milan, Italy) reported the power of combining evolutionary and developmental approaches to study disease-genes such as that encoding the ancestral protein Huntingtin. Expansion over 36 residues of a polymorphic tri-nucleotide CAG repeat, translated into polyglutamine, causes Hungtington Disease (HD), a genetically dominant and fatal neurodegeneration (Zuccato and Cattaneo, 2014). Huntingtin exerts a number of activities in the adult brain, such as promoting the transcription of the neurotrophin BDNF (Zuccato et al., 2001), critical for the survival and activity of the neurons that degenerate in HD. Unexpectedly, huntingtin has also evolved to acquire the unique regulatory activity of controlling neural adhesion by inhibiting ADAM10-mediated processing of *N*-cadherin (Lo Sardo et al., 2012). Besides developmental implications, these observations suggest that ADAM10 might be explored as a new target in the treatment of HD (Lo Sardo et al., 2012).

## Conclusion and Perspectives

Altogether the findings clearly highlighted that metalloproteases are instrumental for proper development of the CNS and of the embryo in general. Most of the basic developmental processes in the CNS involve some type of metalloproteases, which in general exert a beneficial role: they activate pathways that promote neurogenesis, regulate the migration of neurons or the correct growth of axons to their final destinations. Most of the evidence so far indicates that metalloproteases regulate the latter process by degrading axon guidance cues, such as netrin and its receptor DCC or Eph-ephrin molecules (Bai and Pfaff, 2011). Nevertheless, the recent identification of metalloprotease-enriched “invadosomes” in the growth cones of all the neurons so far tested (Santiago-Medina et al., 2015), suggests that metalloproteases might tune other class of molecules such as those involved in focal adhesion. Although not touched upon in this workshop, metalloproteases also cooperate for proper axon myelination and the formation of the extensive vasculature of the brain (Yong et al., 2001). Other developmental events are also likely to require proteases’ function. For example, brain colonization by microglial cell is a poorly explored phenomenon that resemble metastatic invasion, a process in which metalloproteases have a well-established role (Mochizuki and Okada, 2007). Furthermore, microglia cell and astrocytes participate in the regulation of synaptic plasticity and it is quite likely that part of their roles might be mediated by secreted or membrane bound proteases, acting in *cis* or even in *trans*, as mentioned.

Unfortunately, metalloproteases are not always associated to positive events, as their activation or over-activation is often detrimental. As discussed in the workshop, neurodegenerative disorders are a primary example of harmful effect together with metastatic disorders. The role of metalloproteases in these pathologies has been the focus of several laboratories for many years. More recently, general pathological processes such as neuroinflammation have emerged as metalloprotease-regulated events, broadening the implication of these molecules. Thus, it is apparent that we are just observing the tip of the iceberg as we still know little of the physiological relevance of metalloproteases in the brain. Full understanding of their function might unravel how the same protease can have a double edge and might give us some hints on how to turn metalloproteases’ detrimental side into a beneficial activity. Reaching this degree of comprehension requires answering a large list of questions, and we would like to point out some of them, which perhaps represent the most obvious ones (**Table 1**).

**Table 1:** Main open questions in the field of neurobiology linked to protease activities.

What is the portfolio of substrates of a given protease under different conditions?
How is substrate specificity established and modulated?
How does a protease network in a given cell work? How many proteases can use the same substrate and in which sequence?
How can a protease activity be physiologically modulated? What are the protein or lipid factors affecting protease function?
What are the factors affecting protease function in specific tissues or specific developmental processes?
Which signaling processes control intra- and extracellular active proteases?
How do proteases protect themselves from proteolytic activity?
Are proteases suited as therapeutic targets to modulate their activity in the CNS?
Can proteolytical activity be exploited to favor CNS regeneration or neurological diseases?

Likely, more protease activities needs to be identified. The number of substrates each protease can cleave seems to be large. What is the spectrum of substrates used by a specific protease under a given physiological condition? Is there substrate specificity and how is it established? Do proteases compete for the same substrate and how coordination among different proteases is achieved? How is their expression regulated? The physiological modulators of protease activity are also largely unknown: how many are there? Are they specific, likely contributing to define protease/substrate interaction and how do they influence developmental decisions and tissue homeostasis? What is the contribution of ADAMs and MMPs to excitatory and inhibitory synapse formation and homeostasis? How far can proteases act *in trans* in the CNS?

Answering all these questions will certainly lead to a better understanding of metalloproteinase biology but it will require the development of new experimental tools and animal models. For example, there is the need of more sophisticated proteomic approaches, which together with the generation of specific antibodies will be instrumental to fully understand the spectrum of peptides generated by ectodomain shedding or by other types of protease activities. The generation of knock-in mouse mutants with defined mutations in different protease domains should help exploring their precise function and also model human diseases. Furthermore, the recent introduction of new genome editing techniques might be instrumental to tag metalloprotease or their substrates, which could help following proteolytical events and the final destiny of the end products. Most of the work in metalloproteases has been performed in cell lines or mammalian models, but other experimental species, such as the zebrafish, might become handy, especially to tackle metalloprotease implication in developmental events *in vivo*.

When considering the devastating frequency of developmental (autism spectrum disorders) and neurodegenerative (AD and Prion Disease) disorders so far known to be associated to the impaired activity of metalloropeases, it is evident that improving our understanding of metalloprotease biology may have important social benefits, in addition to a scientific relevance. As mentioned above, there is already a considerable effort in exploiting metalloproteases for therapeutics purposes. The task is huge as a protease-targeted therapy will need careful evaluation and it is expected to work only in a tight therapeutic window. Unwanted side effects explained by the modulation of other protease-substrate complexes have to be taken into consideration. Nevertheless, there are considerable expectations that we all hope to foster in the years to come.

## Conflict of Interest Statement

The authors declare that the research was conducted in the absence of any commercial or financial relationships that could be construed as a potential conflict of interest.

## References

[B1] Albright T. D. Jessell T. M. Kandel E. R. Posner M. I. (2000). Neural science: a century of progress and the mysteries that remain. *Cell* 25(Suppl.), S1–S55 10.1016/S0092-8674(00)00251-8 10698132

[B2] Bai G. Pfaff S. L. (2011). Protease regulation: the Yin and Yang of neural development and disease. *Neuron* 72 9–21 10.1016/j.neuron.2011.09.012 21982365 PMC3221598

[B3] Blobel C. P. (2005). ADAMs: key components in EGFR signalling and development. *Nat. Rev. Mol. Cell Biol.* 6 32–43 10.1038/nrm1548 15688065

[B4] Bovolenta P. Esteve P. Ruiz J. M. Cisneros E. Lopez-Rios J. (2008). Beyond Wnt inhibition: new functions of secreted Frizzled-related proteins in development and disease. *J. Cell Sci.* 121 737–746 10.1242/jcs.026096 18322270

[B5] Brennaman L. H. Kochlamazashvili G. Stoenica L. Nonneman R. J. Moy S. S. Schachner M. (2011). Transgenic mice overexpressing the extracellular domain of NCAM are impaired in working memory and cortical plasticity. *Neurobiol. Dis.* 43 372–378 10.1016/j.nbd.2011.04.008 21515372 PMC3129860

[B6] Brennaman L. H. Moss M. L. Maness P. F. (2014). EphrinA/EphA-induced ectodomain shedding of neural cell adhesion molecule regulates growth cone repulsion through ADAM10 metalloprotease. *J. Neurochem.* 128 267–279 10.1111/jnc.12468 24117969 PMC8529540

[B7] Brennaman L. H. Zhang X. Guan H. Triplett J. W. Brown A. Demyanenko G. P. (2013). Polysialylated NCAM and ephrinA/EphA regulate synaptic development of GABAergic interneurons in prefrontal cortex. *Cereb. Cortex* 23 162–177 10.1093/cercor/bhr392 22275477 PMC3513957

[B8] De Groef L. Van Hove I. Dekeyster E. Stalmans I. Moons L. (2014). MMPs in the neuroretina and optic nerve: modulators of glaucoma pathogenesis and repair? *Invest. Ophthalmol. Vis. Sci.* 55 1953–1964 10.1167/iovs.13-13630 24681977

[B9] De Strooper B. (2014). Lessons from a failed ?-Secretase Alzheimer trial. *Cell* 159 721–726 10.1016/j.cell.2014.10.016 25417150

[B10] De Strooper B. Chavez Gutierrez L. (2015). Learning by failing: ideas and concepts to tackle gamma-secretases in Alzheimer’s disease and beyond. *Annu. Rev. Pharmacol. Toxicol.* 55 419–437 10.1146/annurev-pharmtox-010814-124309 25292430

[B11] Doody R. S. Raman R. Farlow M. Iwatsubo T. Vellas B. Joffe S. (2013). A phase 3 trial of semagacestat for treatment of Alzheimer’s disease. *N. Engl. J. Med.* 369 341–350 10.1056/NEJMoa1210951 23883379

[B12] Dziembowska M. Milek J. Janusz A. Rejmak E. Romanowska E. Gorkiewicz T. (2012). Activity-dependent local translation of matrix metalloproteinase-9. *J. Neurosci.* 32 14538–14547 10.1523/JNEUROSCI.6028-11.2012 23077039 PMC6621441

[B13] Emes R. D. Grant S. G. (2012). Evolution of synapse complexity and diversity. *Annu. Rev. Neurosci.* 35 111–131 10.1146/annurev-neuro-062111-150433 22715880

[B14] Endres K. Fahrenholz F. Lotz J. Hiemke C. Teipel S. Lieb K. (2014). Increased CSF APPs-alpha levels in patients with Alzheimer disease treated with acitretin. *Neurology* 83 1930–1935 10.1212/WNL.0000000000001017 25344383

[B15] Esteve P. Sandonis A. Cardozo M. Malapeira J. Ibanez C. Crespo I. (2011). SFRPs act as negative modulators of ADAM10 to regulate retinal neurogenesis. *Nat. Neurosci.* 14 562–569 10.1038/nn.2794 21478884

[B16] Gaublomme D. Buyens T. De Groef L. Stakenborg M. Janssens E. Ingvarsen S. (2014). Matrix metalloproteinase 2 and membrane type 1 matrix metalloproteinase co-regulate axonal outgrowth of mouse retinal ganglion cells. *J. Neurochem.* 129 966–979 10.1111/jnc.12703 24611815

[B17] Jorissen E. Prox J. Bernreuther C. Weber S. Schwanbeck R. Serneels L. (2010). The disintegrin/metalloproteinase ADAM10 is essential for the establishment of the brain cortex. *J. Neurosci.* 30 4833–4844 10.1523/JNEUROSCI.5221-09.2010 20371803 PMC2921981

[B18] Kuhn P. H. Koroniak K. Hogl S. Colombo A. Zeitschel U. Willem M. (2012). Secretome protein enrichment identifies physiological BACE1 protease substrates in neurons. *EMBO J.* 31 3157–3168 10.1038/emboj.2012.173 22728825 PMC3400020

[B19] Lo Sardo V. Zuccato C. Gaudenzi G. Vitali B. Ramos C. Tartari M. (2012). An evolutionary recent neuroepithelial cell adhesion function of huntingtin implicates ADAM10-Ncadherin. *Nat. Neurosci.* 15 713–721 10.1038/nn.3080 22466506

[B20] Marcello E. Gardoni F. Mauceri D. Romorini S. Jeromin A. Epis R. (2007). Synapse-associated protein-97 mediates alpha-secretase ADAM10 trafficking and promotes its activity. *J. Neurosci.* 27 1682–1691 10.1523/JNEUROSCI.3439-06.2007 17301176 PMC6673742

[B21] Marcello E. Saraceno C. Musardo S. Vara H. De La Fuente A. G. Pelucchi S. (2013). Endocytosis of synaptic ADAM10 in neuronal plasticity and Alzheimer’s disease. *J. Clin. Invest.* 123 2523–2538 10.1172/JCI65401 23676497 PMC3668814

[B22] Marcos S. Nieto-Lopez F. Sandonis A. Cardozo M. J. Di Marco F. Esteve P. (2015). Secreted frizzled related proteins modilate pathfinding and fasciculation of mouse retina ganglion cell axons by direct and indirect mechanisms. *J. Neurosci.* 35 4729–4740 10.1523/JNEUROSCI.3304-13.2015 25788689 PMC6605131

[B23] Maretzky T. Mcilwain D. R. Issuree P. D. Li X. Malapeira J. Amin S. (2013). iRhom2 controls the substrate selectivity of stimulated ADAM17-dependent ectodomain shedding. *Proc. Natl. Acad. Sci. U.S.A.* 110 11433–11438 10.1073/pnas.1302553110 23801765 PMC3710827

[B24] Mochizuki S. Okada Y. (2007). ADAMs in cancer cell proliferation and progression. *Cancer Sci.* 98 621–628 10.1111/j.1349-7006.2007.00434.x 17355265 PMC11160018

[B25] Obregon D. Hou H. Deng J. Giunta B. Tian J. Darlington D. (2012). Soluble amyloid precursor protein-alpha modulates beta-secretase activity and amyloid-beta generation. *Nat. Commun.* 3 777 10.1038/ncomms1781 PMC3520614 22491325

[B26] Park S. Lee C. Sabharwal P. Zhang M. Meyers C. L. Sockanathan S. (2013). GDE2 promotes neurogenesis by glycosylphosphatidylinositol-anchor cleavage of RECK. *Science* 339 324–328 10.1126/science.1231921 23329048 PMC3644959

[B27] Pierfelice T. Alberi L. Gaiano N. (2011). Notch in the vertebrate nervous system: an old dog with new tricks. *Neuron* 69 840–855 10.1016/j.neuron.2011.02.031 21382546

[B28] Porlan E. Marti-Prado B. Morante-Redolat J. M. Consiglio A. Delgado A. C. Kypta R. (2014). MT5-MMP regulates adult neural stem cell functional quiescence through the cleavage of N-cadherin. *Nat. Cell Biol.* 16 629–638 10.1038/ncb2993 24952463

[B29] Porlan E. Perez-Villalba A. Delgado A. C. Ferron S. R. (2013). Paracrine regulation of neural stem cells in the subependymal zone. *Arch. Biochem. Biophys.* 534 11–19 10.1016/j.abb.2012.10.001 23073070

[B30] Prox J. Bernreuther C. Altmeppen H. Grendel J. Glatzel M. D’hooge R. (2013). Postnatal disruption of the disintegrin/metalloproteinase ADAM10 in brain causes epileptic seizures, learning deficits, altered spine morphology, and defective synaptic functions. *J. Neurosci.* 33 12915–12928, 12928a. 10.1523/JNEUROSCI.5910-12.2013 23926248 PMC6619719

[B31] Prox J. Willenbrock M. Weber S. Lehmann T. Schmidt-Arras D. Schwanbeck R. (2012). Tetraspanin15 regulates cellular trafficking and activity of the ectodomain sheddase ADAM10. *Cell. Mol. Life Sci.* 69 2919–2932 10.1007/s00018-012-0960-2 22446748 PMC11114675

[B32] Reinhardt S. Schuck F. Grosgen S. Riemenschneider M. Hartmann T. Postina R. (2014). Unfolded protein response signaling by transcription factor XBP-1 regulates ADAM10 and is affected in Alzheimer’s disease. *FASEB J.* 28 978–997 10.1096/fj.13-234864 24165480

[B33] Rodriguez J. Esteve P. Weinl C. Ruiz J. M. Fermin Y. Trousse F. (2005). SFRP1 regulates the growth of retinal ganglion cell axons through the Fz2 receptor. *Nat. Neurosci.* 8 1301–1309 10.1038/nn1547 16172602

[B34] Romi E. Gokhman I. Wong E. Antonovsky N. Ludwig A. Sagi I. (2014). ADAM metalloproteases promote a developmental switch in responsiveness to the axonal repellant Sema3A. *Nat. Commun.* 5 4058 10.1038/ncomms5058 24898499

[B35] Rushworth J. V. Griffiths H. H. Watt N. T. Hooper N. M. (2013). Prion protein-mediated toxicity of amyloid-beta oligomers requires lipid rafts and the transmembrane LRP1. *J. Biol. Chem.* 288 8935–8951 10.1074/jbc.M112.400358 23386614 PMC3610967

[B36] Sabharwal P. Lee C. Park S. Rao M. Sockanathan S. (2011). GDE2 regulates subtype-specific motor neuron generation through inhibition of Notch signaling. *Neuron* 71 1058–1070 10.1016/j.neuron.2011.07.028 21943603 PMC3183458

[B37] Santiago-Medina M. Gregus K. A. Nichol R. H. O’toole S. M. Gomez T. M. (2015). Regulation of ECM degradation and axon guidance by growth cone invadosomes. *Development* 142 486–496 10.1242/dev.108266 25564649 PMC4302990

[B38] Saraceno C. Marcello E. Di Marino D. Borroni B. Claeysen S. Perroy J. (2014). SAP97-mediated ADAM10 trafficking from Golgi outposts depends on PKC phosphorylation. *Cell Death Dis.* 5 e1547. 10.1038/cddis.2014.492 PMC4260750 25429624

[B39] Sudhof T. C. (2008). Neuroligins and neurexins link synaptic function to cognitive disease. *Nature* 455 903–911 10.1038/nature07456 18923512 PMC2673233

[B40] Suzuki K. Hayashi Y. Nakahara S. Kumazaki H. Prox J. Horiuchi K. (2012). Activity-dependent proteolytic cleavage of neuroligin-1. *Neuron* 76 410–422 10.1016/j.neuron.2012.10.003 23083742

[B41] Taylor D. R. Parkin E. T. Cocklin S. L. Ault J. R. Ashcroft A. E. Turner A. J. (2009). Role of ADAMs in the ectodomain shedding and conformational conversion of the prion protein. *J. Biol. Chem.* 284 22590–22600 10.1074/jbc.M109.032599 19564338 PMC2755666

[B42] Tsilibary E. Tzinia A. Radenovic L. Stamenkovic V. Lebitko T. Mucha M. (2014). Neural ECM proteases in learning and synaptic plasticity. *Prog. Brain Res.* 214 135–157 10.1016/B978-0-444-63486-3.00006-2 25410356

[B43] van der Kooij M. A. Fantin M. Rejmak E. Grosse J. Zanoletti O. Fournier C. (2014). Role for MMP-9 in stress-induced downregulation of nectin-3 in hippocampal CA1 and associated behavioural alterations. *Nat. Commun.* 5 4995 10.1038/ncomms5995 PMC4199199 25232752

[B44] Van Hove I. Verslegers M. Hu T. T. Carden M. Arckens L. Moons L. (2015). A proteomic approach to understand MMP-3-driven developmental processes in the postnatal cerebellum: chaperonin CCT6A and MAP kinase as contributing factors. *Dev. Neurobiol.* 10.1002/dneu.22272 [Epub ahead of print] 25652596

[B45] Weber S. Saftig P. (2012). Ectodomain shedding and ADAMs in development. *Development* 139 3693–3709 10.1242/dev.076398 22991436

[B46] Yong V. W. Power C. Forsyth P. Edwards D. R. (2001). Metalloproteinases in biology and pathology of the nervous system. *Nat. Rev. Neurosci.* 2 502–511 10.1038/35081571 11433375 PMC7097548

[B47] Zipursky S. L. Sanes J. R. (2010). Chemoaffinity revisited: dscams, protocadherins, and neural circuit assembly. *Cell* 143 343–353 10.1016/j.cell.2010.10.009 21029858

[B48] Zuccato C. Cattaneo E. (2014). Huntington’s disease. *Handb. Exp. Pharmacol.* 220 357–409 10.1007/978-3-642-45106-5_14 24668480

[B49] Zuccato C. Ciammola A. Rigamonti D. Leavitt B. R. Goffredo D. Conti L. (2001). Loss of huntingtin-mediated BDNF gene transcription in Huntington’s disease. *Science* 293 493–498 10.1126/science.1059581 11408619

